# Distinct commensal bacteria in human nasopharyngeal lymphoid tissue associated with localized immunological memory

**DOI:** 10.1016/j.isci.2025.114579

**Published:** 2025-12-30

**Authors:** Seung-Taek Park, Jina Won, Siyeon Jin, Sujin Kim, Haeun Shin, Su Hyun Lim, Ye-Ji Bang, Hyun Jik Kim

**Affiliations:** 1 Department of Biomedical Sciences, Seoul National University College of Medicine, Seoul, Korea; 2 Department of Otorhinolaryngology, Seoul National University College of Medicine, Seoul, Korea; 3 Department of Otorhinolaryngology, Seoul National University Hospital, Seoul, Korea; 4 Department of Microbiology and Immunology, Seoul National University College of Medicine, Seoul, Korea; 5 Institute of Endemic Diseases, Seoul National University Medical Research Center, Seoul, Korea; 6 Sensory Organ Research Institute, Seoul National University Medical Research Center, Seoul, Korea

**Keywords:** immunology, microbiology

## Abstract

The nasopharynx (NP) serves as a primary site for localized immune responses that restrict the spread of SARS-CoV-2 to the lower respiratory tract. The microbiome is increasingly recognized as a key modulator of antiviral immunity but whether it shapes immune responses in upper airway remains uncharacterized. Detailed microbial profiles revealed that *S*. *aureus* complex abundance was the primary discriminating factor of microbial community in the NP and the enhanced abundance of *S*. *aureus* complex correlated with higher frequencies of CD4^+^, CD8^+^ tissue-resident memory T (T_RM_), and B_RM_ cells. The abundance of *S*. *aureus* complex was closely associated with distinct metabolic pathways, particularly those involved in nitrogen metabolism (e.g., arginine, ornithine, and proline interconversion) and the mevalonate pathway for carotenoid biosynthesis. These findings suggest that *S*. *aureus* complex may foster unique metabolic dynamics in the NP in enhancing the tissue-residency of memory cells and localized immune responses in upper airway.

## Highlights

• *S*. *aureus* complex are dominant bacterial species in human NP mucosa
• *S*. *aureus* complex are the key discriminating factors in NP microbial community
• The abundance of *S*. *aureus* complex correlates with the frequencies of T_RM_ and B_RM_
• *S*. *aureus* complex-induced metabolites regulate the tissue residency of immune cells

## Graphical abstract

**Figure:**
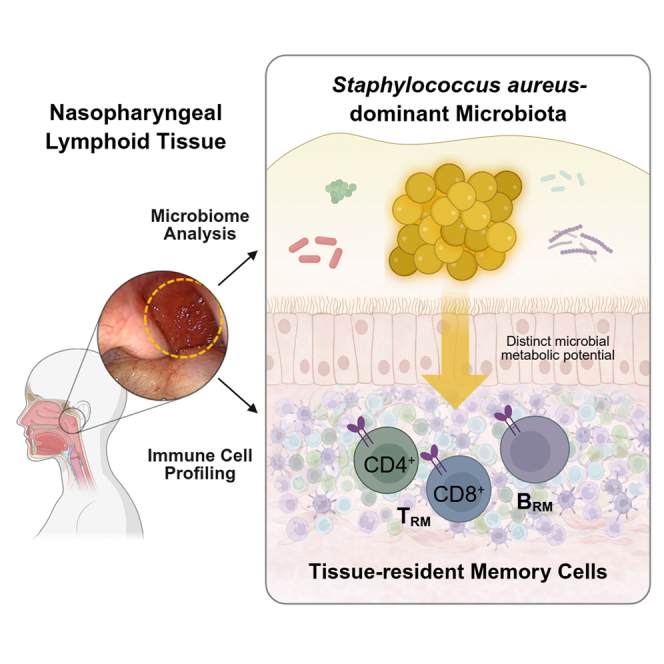

## Introduction

Multiple datasets have identified putative severe acute respiratory syndrome coronavirus 2 (SARS-CoV-2) targets in the upper airway, including the nasal mucosa and nasopharyngeal (NP) lymphoid tissue (adenoid). After infection begins in the upper airway, viral spread progresses to the respiratory tract unless appropriate activation of immune mechanisms prevents it.^1^^,^^2^^,^^3^ Although not all patients diagnosed with coronavirus disease 2019 (COVID-19) progress to severe lung disease, failed antiviral immunity in the upper airway underlies and precedes severe COVID-19.^4^^,^^5^^,^^6^ Therefore, a better understanding of immune responses in the upper airway could provide insights into the efficient defense mechanisms needed to restrict the spread of SARS-CoV-2 at the initial stage of infection. Although peripheral blood has been shown to be important for sustained immune responses after vaccination or SARS-CoV-2 infection, virus-host encounters take place in NP lymphoid tissue, and balanced antiviral properties in the NP are essential to protect the respiratory tract from viral infection.^3^^,^^5^^,^^7^^,^^8^

Virus-specific memory CD4^+^ and CD8^+^ T and CD19^+^ B cells compose heterogenous subsets of circulating cells and tissue-resident memory (T_RM_ and B_RM_) cells in various sites.^9^ We speculate that NP lymphoid tissue is a challenging site for immunological memory as a frontline immune structure in the upper airway that is central to the protective immunity provided by vaccines and direct infections. Pioneering work noted a distribution of SARS-CoV-2-induced memory T and B cells in NP lymphoid tissues.^10^^,^^11^ However, localized alteration of T_RM_ and B_RM_ populations in the NP are largely unexplored, and critical outstanding questions remain about the regulatory mechanisms associated with their abundance, and residency in NP lymphoid tissue.

Many studies have demonstrated that mucosal microbiota engage in complex interactions with the host to maintain balance with the host immunity.^12^^,^^13^^,^^14^^,^^15^ Importantly, studies on the reaction of the colonizing microbiome in the respiratory mucosa to an inhaled virus increasingly consider the contribution of virus restriction at the initial stage of infection.^16^^,^^17^ We found that the upper half of NP lymphoid tissue contains respiratory mucosa including epithelium.^7^ Therefore, we assume that the endogenous microbiome presents at the NP mucosal surface and that the localized immune cell population might be associated with NP microbiome.

In this work, we examined the association between immunological memory diversity and alterations in NP microbial composition according to COVID-19 infection or vaccination and sought to elucidate the local coordination of the NP microbiome and cellular and humoral immune responses, especially tissue-resident memory cells. We identified a distinct correlation between prevalent commensal bacteria, *Staphylococcus aureus* complex and the frequencies of CD4^+^ T_RM_, CD8^+^ T_RM_, B_RM_, and plasmablast in NP lymphoid tissues. Our results also suggest that *S*. *aureus* complex might strongly affect the landscape of tissue-resident memory cells through both metabolic and immunological mechanisms.

## Results

### Demographics data of subjects included in the study

**Figure 1:**
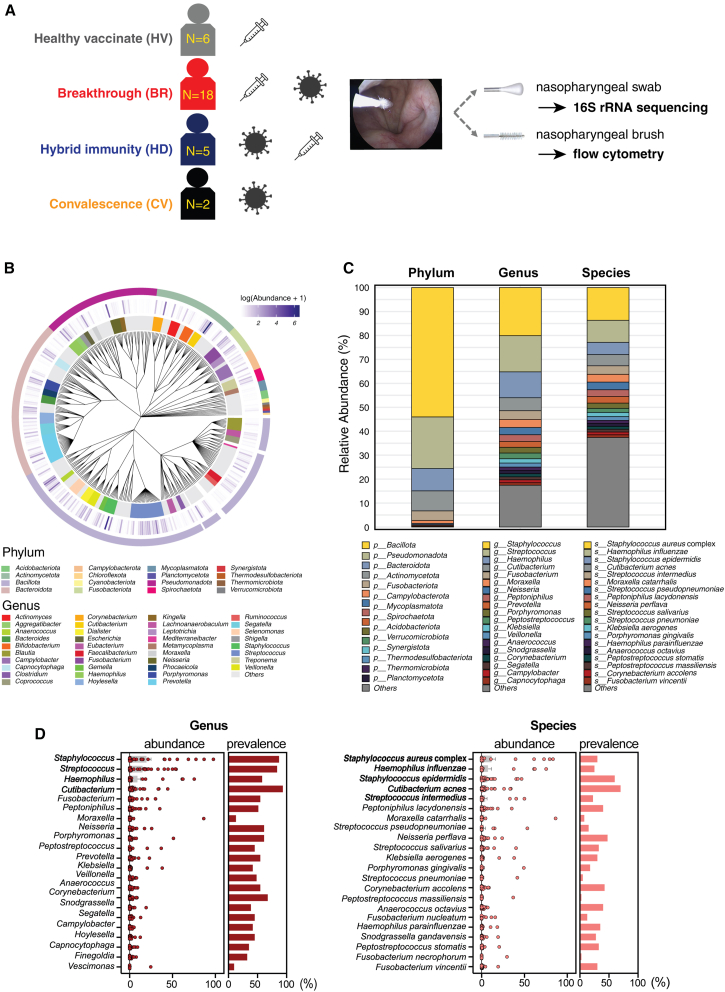
Microbiome diversity and composition in the NP (A) Schematic representation of the study groups, healthy vaccinated (HV, *n* = 6), breakthrough (BR, *n* = 18), hybrid immunity (HD, *n* = 5), and convalescence (CV, *n* = 2). The sampling methods and analyses performed are indicated. (B) Phylogenetic tree showing the taxonomic relationships and relative abundances of bacterial taxa. The inner ring represents genera, and the outer ring represents phyla. Color intensity indicates log-transformed abundance. (C) Stacked bar plots showing the relative abundances at the phylum, genus, and species levels across all samples. (D) Abundance and prevalence of dominant bacterial taxa. Left:, genus level; right: species level. Dot plots show the relative abundance (%) in individual samples. Bar graphs indicate prevalence (%) across all samples. Taxa are ordered by mean relative abundance.

We compared the frequency of immune cells and the microbial compositions in paired NP brushings and swab samples in health vaccinated (HV, *N* = 6), breakthrough (BR, *N* = 18), hybrid immunity (HD, *N* = 5), and convalescent (CV, *N* = 2) donors (Figure 1A and Video S1). We collected demographic data and the baseline characteristics of the recruited subjects are shown in Table 1. Subjects received at least 1–3 doses of COVID-19 vaccines, except for two CV donors who underwent NP swabs and brushings an average of 23.6 months after COVID-19 diagnosis. HV donors received nasal swabs and brushings on average 21.1 months after the last vaccination, BR donors on average 12.47 months after COVID-19 confirmation following vaccinations, and HD patients on average 15.4 months after the third vaccination following COVID-19 diagnosis. The HV, BR, and HD subjects did not exhibit any statistically significant differences in their clinical parameters except their mean age. Using this cohort, we examined alterations in immune cell frequencies and microbial communities in the NP.

**Table 1:** ^1^Baseline characteristics of the recruited subjects

	HV	BR	HD	CV	*p*-value
Patient number (n)	6	18	5	2	–
Mean age (years old)	27	46.1	49.6	33	*0*.*01*
Sex (M:F)	6:0	14:4	4:1	1:1	–
Mean body mass index (kg/m^2^)	22.4	21.8	21.5	*22*.*7*	–
Number of vaccinations	2.67	2.56	2.60	*NA*	*0*.*99*

**Types of vaccines (n = patient number)**					

AstraZeneca	0	2	0	0	–
Pfizer	4	11	2	0	
Moderna	2	4	3	0	
Janssen	0	1	0	0	
Time from last vaccination/infection (months)	21	12.47	15.4	*NA*	*0*.*368*

^1^Group comparisons were performed using the Kruskal–Wallis test across the four groups (HV, BR, HD, CV). *p*-values denote statistical significance at two-sided *p* < 0.05.

### Characterization of microbial communities in NP lymphoid tissue

First, we performed unbiased bacterial 16S rRNA sequencing to characterize the microbial communities in the NPs of healthy subjects (*N* = 31). The full-length 16S rRNA amplicons (V1–V9 region) were sequenced, and 2,587 amplicon sequence variants (ASVs) representing 379 total species were identified. The phylum-level analysis demonstrated that *Bacillota* commensal bacteria were most enriched in the NP, and *Pseudomonadota* and *Bacteroidota* were also dominant microbial phyla. We found that *Staphylococcus*, *Streptococcus*, *Haemophilus*, and *Cutibacterium* were the dominant genera in the NP. At the species level, *Haemophilus influenzae*, *Staphylococcus epidermidis*, *Cutibacterium acnes*, and *Streptococcus intermedius* were enriched (Figures 1B and 1C), with *S*. *aureus* complex showing the highest mean relative abundance (13.7%) across samples. Although the dominant commensal bacteria all showed high abundance and prevalence in the NP, their distribution varied significantly among individuals (Figure 1D).

Species-level classification using 16S rRNA gene sequencing is often limited, particularly within genera such as *Staphylococcus*, *Streptococcus*, and *Escherichia/Shigella*, due to high sequence similarity among closely related species.^18^ In our dataset, certain ASVs aligned with high identity (≥99.4%) to multiple members of the *S. aureus* complex—including *S*. *aureus*, *S*. *roterodami*, *S*. *argenteus*, and *S*. *schweitzeri*—with less than 0.2% difference in similarity between the top-scoring species. Due to the limited discriminatory power of 16S rRNA sequences among these species, even with the full-length 16S sequencing, these ASVs were conservatively grouped and annotated as the *S. aureus* complex.^19^

To further resolve species-level identity within the *S*. *aureus* complex, we performed shotgun metagenomic sequencing on five NP samples (HV4, BR1, BR2, BR17, and BR18) that exhibited the highest relative abundance of the *S*. *aureus* complex. Although high levels of host DNA contamination precluded metagenome-assembled genome (MAG) construction, direct mapping of host-filtered reads to reference genomes of *S*. *aureus*, *S*. *roterodami*, *S*. *argenteus*, and *S*. *schweitzeri* revealed that over 95% of species-specific reads matched *S*. *aureus* (Figures S1A and S1B). These findings support *S*. *aureus* as the predominant species within the complex in our cohort.

**Figure 2:**
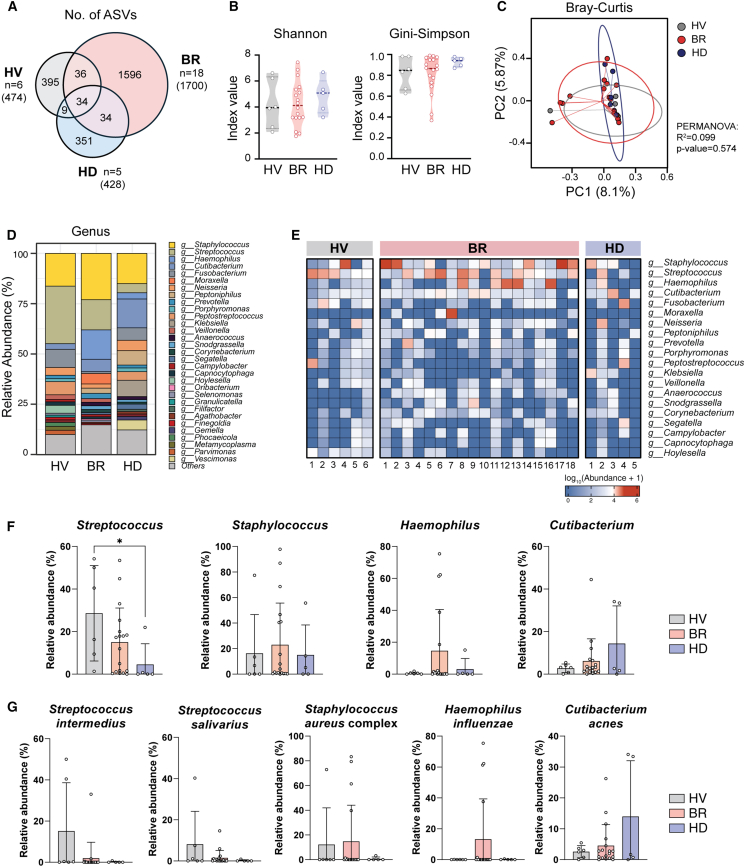
Microbiome diversity and composition comparison among the HV, BR, and HD groups (A) Venn diagram showing the distribution of unique and shared amplicon sequence variants (ASVs) among the three groups. Numbers indicate the count of ASVs in each section, with total number of ASVs per group shown in parentheses. (B) Alpha diversity measures. Left: Shannon diversity index. Right, Gini-Simpson index. Each dot represents an individual sample, with violin plots showing the distribution of values for each group. (C) Beta diversity analysis calculated using Bray-Curtis dissimilarity and visualized through a principal coordinate analysis (PCoA). Each point represents a sample, color-coded by group (HV, BR, and HD). Ellipses indicate 95% confidence intervals for each group. The PERMANOVA results (R2 and *p* value) are provided. (D) Stacked bar chart showing the relative abundances of the top 30 bacterial genera in each group (HV, BR, and HD). Colors represent different genera, with the legend provided to the right. (E) Heatmap displaying the log10-transformed abundances of key bacterial genera across individual samples in each group. Columns represent samples, rows represent genera, and color intensity indicates abundance (blue, low; red, high). Comparison of microbial composition in HV, BR, and HD groups at the (F) genus- and (G) species-levels. (Data are represented as mean ± standard deviation [SD]: Kruskal-Wallis test followed by Dunn’s multiple-comparison test). ∗*p* < 0.05.

We divided the recruited subjects into three groups (HV [*n* = 6], BR [*n* = 18], HD [*n* = 5]) and compared their NP microbial communities. The CV group, which had only two donors, was excluded from the comparative analysis. ASV-level analysis revealed 34 core ASVs shared across all groups, with BR donors contributing the highest number of unique ASVs (*n* = 1,700) compared to HV (*n* = 474) and HD (*n* = 428) (Figure 2A). Species-level analysis similarly identified 88 core species, with a greater number of unique species observed in the BR group—though this may in part reflect its larger sample size (Figures 2A, S2A, and S2B). Analysis of α-diversity (Shannon and Gini-Simpson diversity indices) showed no significant difference among the HV, BR, and HD donors (Figure 2B). The PCoA based on the Bray-Curtis distance matrix suggested that the richness of the microbial communities (β-diversity) was not clearly divided according to the history of COVID-19 infection or vaccination (Figure 2C). While neither COVID-19 infection nor vaccination history altered the evenness or richness of the NP microbiome, we determined the microbial composition in the NPs of healthy subjects at the species level and *S*. *aureus* complex is the predominant species within the complex of NP microbiome.

### NP microbiome composition depending on COVID-19 infection and vaccination

Although analysis of alpha- and beta-diversity metrics revealed no significant differences between groups, detailed taxonomic analysis revealed intriguing abundance patterns of the four predominant genera - *Streptococcus*, *Staphylococcus*, *Haemophilus*, and *Cutibacterium* - across HV, BR, and HD groups (Figures 2D–2F). *Streptococcus* was the most abundant genus in the NPs of HV donors, and its distribution was reduced in BR and HD donors. The distribution of *Staphylococcus* was relatively higher and *Haemophilus* was also abundant in the NP of BR donors. The number of subjects was small, but *Cutibacterium* was particularly highly abundant in the NPs of HD donors.

Our species-level analysis of the NP microbiomes revealed that *Streptococcus intermedius* (HV1, HV3) and *Streptococcus salivarius* (HV2) were associated with *Streptococcus* genus abundance in the NPs of HV donors. While just one HV donor (HV4) showed a high abundance of *S. aureus* complex, this complex was the most dominant species in the NPs of BR donors (BR1, BR2, BR17, and BR18). In all the BR donors whose most prevalent genus in the NP was *Haemophilus*, *H*. *influenzae* was relatively abundant species (BR8, BR12, BR13, and BR16). We also found that *C*. *acnes* was dominant *Cutibacterium* species in the NPs of HD donors (HD3, HD5) (Figure 2G, S3A, and S3B).

### Classification of NP microbiome according to the abundance of *S*. *aureus* complex

**Figure 3:**
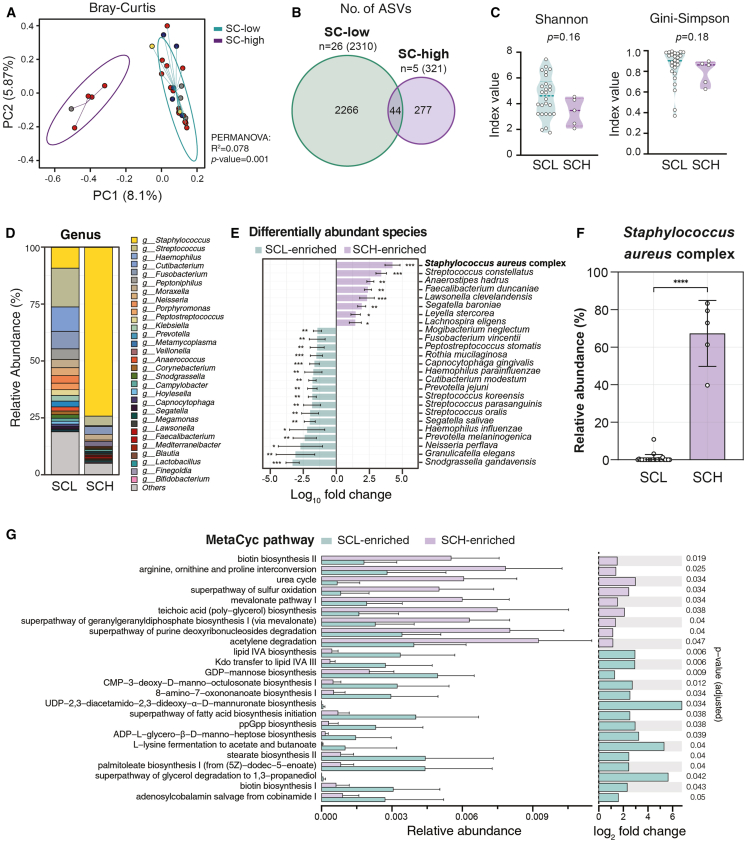
Microbiome diversity and composition analysis in the SC-low vs. SC-high groups (A) Beta diversity analysis calculated using the Bray-Curtis dissimilarity and visualized through a principal coordinate analysis (PCoA). Each point represents a sample, color-coded by group (SC-low in teal, SC-high in purple). Ellipses indicate 95% confidence intervals for each group. The PERMANOVA results (R^2^ = 0.078, *p* value = 0.001) are provided. (B) Venn diagram showing the distribution of unique and shared amplicon sequence variants (ASVs) between the SC-low and SC-high groups. Numbers indicate the ASVs in each section, with total ASVs per group shown in parentheses. (C) Alpha diversity measures. Left, Shannon diversity index; right, Gini-Simpson index. Each dot represents an individual sample, with violin plots showing the distribution of values in each group. *p* values for group comparisons are provided (Shannon, *p* = 0.16, Gini-Simpson, *p* = 0.18). (D) Stacked bar chart showing the relative abundance of bacterial genera in the SC-low (SCL) and SC-high (SCH) groups. Colors represent different genera, with the legend provided to the right. (E) Differentially abundant species identified by the ANCOM-BC II analysis. The *x* axis shows the log-fold change, with species enriched in SCL (teal) or SCH (purple). ∗*p* < 0.05, ∗∗*p* < 0.01, ∗∗∗*p* < 0.001. (F) Bar plot showing the relative abundance of *Staphylococcus aureus* complex in the SCL and SCH groups (data are represented as mean ± SD). The *y* axis shows relative abundance (%). ∗∗∗∗*p* < 0.0001, Wilcoxon rank-sum test. (G) MetaCyc pathways enriched in the SCL and SCH groups, showing the relative abundances and log2 fold changes.

Following our observation that the NP microbiome samples clustered into two distinct groups independent of COVID-19 infection or vaccine status (PERMANOVA, PC1 8.1%, PC2 5.87%, R^2^ = 0.078, *p* = 0.001; Figures 3A and S4A), we investigated the key factors driving this natural separation. Through a detailed analysis of microbial compositions, we identified *S*. *aureus* complex abundance as the primary discriminating factor and classified the samples into *S*. *aureus* complex-low (SCL, *n* = 26) and *S*. *aureus* complex-high (SCH, *n* = 5, HV4, BR1, BR2, BR17, and BR18) groups (Table 2). Between the groups, 2310 ASVs were unique to SCL, and 321 ASVs were unique to SCH, with 44 ASVs shared between the groups (Figure 3B). Although no statistically significant differences were found, the alpha diversity metrics showed a trend toward lower microbial diversity in the SCH group (Figure 3C).

**Table 2:** ^1^Baseline characteristics of SCL and SCH donors

	SCL	SCH	*p* value
Patient number (n)	26	5	–
Mean age (years old)	42.12	42.20	*0*.*97*
Sex (M:F)	22:4	4:1	–
Number of vaccinations	2.42	2.4	*0*.*90*

**Types of vaccines (n = patient number)**			

AstraZeneca	1	1	–
Pfizer	14	3	
Moderna	9	0	
Janssen	0	1	
Time from last vaccination/infection (months)	15.36	13.80	*0*.*93*

^1^Group comparisons were performed using the Wilcoxon rank-sum test. *p*-values denote statistical significance at two-sided *p* < 0.05.

A detailed examination of individual samples revealed that this classification reflected consistent patterns at both the genus and species levels, with SCH samples showing a marked dominance of *Staphylococcus* (genus) and *S*. *aureus* complex (species), and SCL samples displaying more heterogeneous bacterial compositions (Figures 3D, S4B, and S4C). The ANCOM-BC II analysis identified distinct species enrichment patterns between the groups (Figure 3E). Microbial signatures were further validated by applying the random forest classification to check for overlapped features. Among the species with significant importance, three of them overlapped with the species selected in the ANCOM-BC II analysis: *S*. *aureus* complex, *Neisseria perflava*, and *Faecalibacterium duncaniae* (Figure S5A). An analysis of relative abundance showed a significant difference only for *S*. *aureus* complex (Figures 3F and S5B), confirming its role as the key determinant of this natural microbial community stratification.

MetaCyc pathway analysis based on 16S rRNA sequencing data predicted distinct microbial metabolic capabilities between SCL and SCH groups (Figure 3G). Twenty-four metabolic pathways showed distinctive enrichment between groups, with specific bacterial metabolic potentials analyzed for each pathway (Figure S6). Among these, pathways involved in nitrogen metabolism—specifically the arginine, ornithine and proline interconversion pathway and the urea cycle—showed significant enrichment in the SCH group, suggesting potential differences in amino acid metabolism and nitrogen processing. Previous study shows that Intracellular L-arginine levels regulate several metabolic pathways in T cells and T cells with increased L-arginine display enhanced survival.^20^ These metabolomic and proteomic profiling unveil intracellular L-arginine as a crucial regulator of metabolic fitness, survival capacity, and activity of memory T cells. Notably, the SCH group also showed significant enrichment of the mevalonate pathway I, a crucial bacterial pathway for carotenoid biosynthesis.^21^ These findings are particularly significant because *S*. *aureus* complex may activate arginine-related metabolic dynamics and provide carotenoid precursors that contribute to local retinoid metabolism in NP, which plays a fundamental role in tissue-residency and T cell survival in barrier tissues.^22^^,^^23^ These predicted metabolic pathways highlight the potential role of *S*. *aureus* complex as a key bacterial species that may shape the immune microenvironment of the NP.

### Immune cell population associated with the abundance of *S*. *aureus* complex

**Figure 4:**
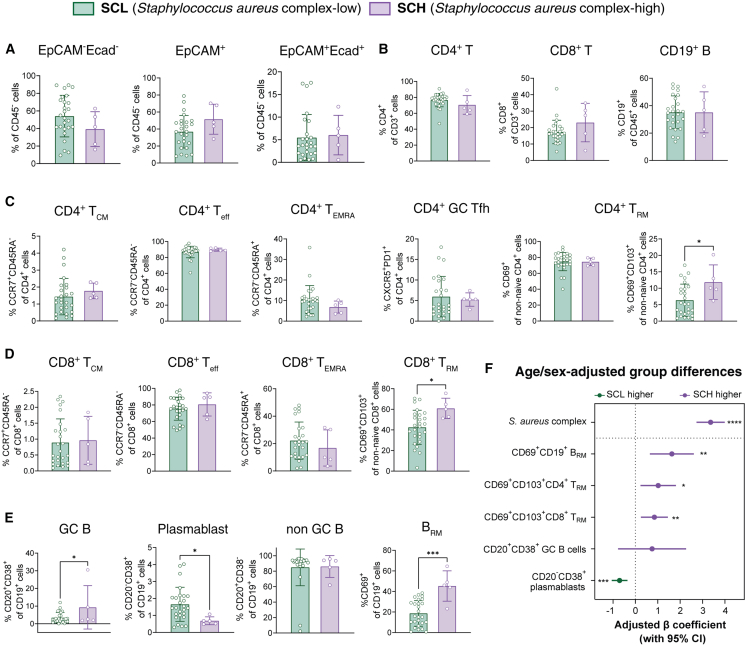
The frequencies of CD45^+^ and CD45^−^ cells depending on *S*. *aureus* complex abundance Differences in immune cell frequencies, ratios, and subsets in the NPs of the SCH (*n* = 5) and SCL (*n* = 19) groups by flow cytometry. (A) Percentage of CD45^−^ epithelial cells (EpCAM^−^Ecad^−^/EpCAM^+^Ecad^−^/EpCAM^+^Ecad^+^). (B) Percentage of CD4^+^, CD8^+^ T cells (of CD3^+^ cells) and CD19^+^ B cells (of CD45^+^ cells). (C) Percentages of T_CM_ (CCR7^+^CD45RA^−^), T_eff_ (CCR7^−^CD45RA^−^), T_EMRA_ (CCR7^−^CD45RA^+^), GC Tfh (CXCR5^+^PD-1^+^), CD69^+^ T_RM_, and CD69^+^CD103^+^ T_RM_ cells among all CD4^+^ T cells. (D) Percentages of T_CM_ (CCR7^+^CD45RA^−^), T_eff_ (CCR7^−^CD45RA^−^), T_EMRA_ (CCR7^−^CD45RA^+^), and T_RM_ (CD69^+^CD103^+^) cells among all CD8^+^ T cells. (E) Percentages of GC B cells (CD20^+^CD38^+^), antibody-secreting cells (CD20^−^CD38^+^), non-GC B cells (CD20^+^CD38^−^), and CD69^+^ B_RM_ cells among all B cells (data are represented as mean ± SD: Wilcoxon rank-sum test). (F) Age- and sex-adjusted group differences in microbial and immune features. Forest plot shows β coefficients (±95% CI) from multivariable linear regression models assessing the association of the SCH group with each feature after adjustment for age and sex. The relative abundance of the *S*. *aureus* complex was log-transformed, and immune-cell frequencies were logit-transformed. ∗*p* < 0.05, ∗∗*p* < 0.01, ∗∗∗*p* < 0.001, ∗∗∗∗*p* < 0.0001.

We integrated the microbial profiles with the proportion of immune cells in the NP according to the abundance of *S*. *aureus* complex to uncover the correlation between predominant microbial abundance and the frequency of localized memory immune cells. The flow cytometry data present the frequency of epithelial cells (CD324 and CD326), CD4^+^ or CD8^+^ circulating memory T cells (CD45RA and CCR7), CD4^+^ CD8^+^ T_RM_ (CD103 and CD69) cells, CD4^+^ Tfh (PD-1 and CXCR5) cells, CD19^+^ B cell subtypes (CD20 and CD38), and B_RM_ (CD19 and CD69) cells (Figure S7). Although the frequency of lymphocytes present in the NP did not differ among the HV, BR, and HD donors (Figures S8A and S8B), we found an interesting difference in the subtypes of CD4^+^, CD8^+^ T cells, and CD19^+^ B cells between the SCH and SCL groups (Figure 4).

The flow cytometry data reveal that the proportions of CD324^+^ and CD326^+^ epithelial cells in the NP did not differ between the SCH and SCL groups (Figure 4A). The frequencies of CD45^+^ lymphocytes in the NP did not differ depending on *S*. *aureus* complex abundance either (Figure 4B). In addition, the proportions of memory CD4^+^ T cells (CD4^+^ T_CM_, CD4^+^ T_eff_, CD4^+^ T_EMRA_, and GC Tfh cells) in the NP did not differ whether *S*. *aureus* complex was abundant in the NP or not (Figure 4C). However, the frequency of CD69^+^CD103^+^CD4^+^ T_RM_ cells in the NP was significantly higher in the SCH group. Interestingly, the frequency of CD69^+^CD103^+^CD8^+^ T_RM_ cells in the NP was also higher in the SCH group (Figure 4D). In addition, the frequencies of both GC B cells and B_RM_ (CD19^+^CD69^+^) cells in the NP were higher in donors with more *S*. *aureus* complex (Figure 4E). To ensure that these associations were independent of demographic variables, we performed a multivariable linear regression analysis adjusting for age and sex (Figure 4F). The enrichment of the *S*. *aureus* complex in the SCH group remained highly significant after adjustment. Similarly, the increased frequencies of CD69^+^CD103^+^CD4^+^ T_RM_ cells, CD69^+^CD103^+^CD8^+^ T_RM_ cells, and B_RM_ (CD69^+^CD19^+^), along with a decrease in plasmablasts, remained significantly associated with the SCH group, while the difference in GC B cells was not significant after adjustment. These results demonstrate that the abundance of *S*. *aureus* complex, might be associated with distinct immune cell changes in NP lymphoid tissue, especially the enhancement of tissue-resident memory cells.

### Distinctive localized immune cell population in BR and HD donors depending on abundant NP microbial species

**Figure 5:**
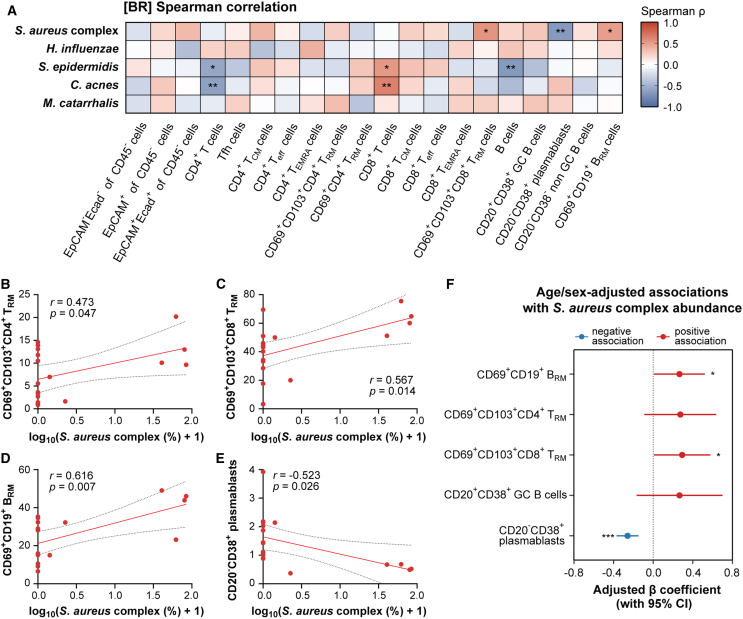
Correlation and adjusted associations between microbial species and the immune cell population in the NPs of BR donors (A) A heatmap of the Spearman correlations between major microbial species and the percentage of immune cells (*n* = 19). (B–E) Scatterplots showing correlations between *S*. *aureus* complex abundance and the indicated immune-cell subsets: (B) CD69^+^CD103^+^CD4^+^ T_RM_, (C) CD69^+^CD103^+^CD8^+^ T_RM_, (D) CD69^+^CD19^+^ B_RM_, and (E) CD20^−^CD38^+^ plasmablasts. Red line = linear fit; dashed line = 95% CI. Pearson correlation coefficients (*r*) and *p* values are shown. (F) Age- and sex-adjusted associations between *S*. *aureus* complex abundance and immune-cell subsets. Forest plot shows β coefficients (±95% CI) from multivariable linear regression models, assessing the association between log-transformed *S*. *aureus* complex abundance (continuous predictor) and logit-transformed immune-cell frequencies, adjusted for age and sex. ∗*p* < 0.05, ∗∗*p* < 0.01.

The 16S rRNA bacterial profile data show that *S*. *aureus* complex was significantly more abundant in BR donors (BR1, BR2, BR17, and BR18) than in HV or HD donors, and the composition of *S*. *epidermidis* was also high in two of BR donors (BR10 and BR15). We investigated the relationship between the population of immune cells in the NPs of BR donors and the commensal microbial abundance of *S*. *aureus* complex and *S*. *epidermidis* (Figure 5A). Based on Spearman correlation analysis, we found that the SCH group showed significantly higher frequencies of CD69^+^CD103^+^CD8^+^ T_RM_, and B_RM_ cells than the SCL group. In contrast, the frequency of plasmablasts was negatively associated with the abundance of *S*. *aureus* complex (Figure 5A). Furthermore, the abundance of *S*. *epidermidis* in the NPs of BR donors was positively correlated with CD8^+^ T cells, but showed negative correlations with both CD4^+^ T cells and B cells.

Univariate linear regression analysis revealed a significant positive linear relationship between the abundance of the *S*. *aureus* complex and the frequencies of tissue-resident memory cells. Specifically, higher *S*. *aureus* complex abundance was linearly associated with increased frequencies of CD69^+^CD103^+^CD4^+^ T_RM_ (r = 0.473, *p* = 0.047), CD69^+^CD103^+^CD8^+^ T_RM_ (r = 0.567, *p* = 0.014), and CD69^+^CD19^+^ B_RM_ cells (r = 0.616, *p* = 0.007) in the NPs (Figures 5B–5D). In contrast, a significant negative linear relationship was observed with the population of CD19^+^CD20^−^CD38^+^ plasmablasts (r = −0.523, *p* = 0.026) (Figure 5E). To control for potential confounding effects of age and sex, we next performed a multivariable linear regression. In this adjusted model, the relationships with CD69^+^CD103^+^CD8^+^ T_RM_, B_RM_ cells, and plasmablasts remained significant. Although the association with CD69^+^CD103^+^CD4^+^ T_RM_ did not reach statistical significance (*p* = 0.128), it continued to show a positive trend.

The HD donors showed distinct microbial communities in their NPs, with *Cutibacterium* (genus) and *C*. *acnes* (species) as the most abundant microbes (Figures 2D and S3A). We investigated the relationship between the population of immune cells and the abundance of *C*. *acnes* (Figure S9). HD donors with a higher abundance of *C*. *acnes* tended to have lower frequencies of CD4^+^CXCR5^+^PD-1^+^ Tfh, CD20^+^CD38^+^ GC B, and CD69^+^CD19^+^ B_RM_ cells. In contrast, a higher abundance of *C*. *acnes* was associated with an increased frequency of CD8^+^ T_CM_ cells. These data reveal an intriguing association between NP microbial species and the alteration of immunological memory cells, especially CD4^+^, CD8^+^ T_RM_, B_RM_, and plasmablast in the NPs of BR and HD donors.

## Discussion

In this study, we establish the distinct relation between the distribution of certain commensal bacteria and the frequencies of localized immune memory cells in the NP mucosa. In particular, the abundance of *S*. *aureus* complex might be associated with the frequency of immune memory cells, especially tissue-resident memory T and B cells, GC B cells, and plasmablasts in NP lymphoid tissue following SARS-CoV-2 infection and vaccination. The current data could help uncover the site-directed coordination of cellular and humoral immunity and *S*. *aureus* complex-related metabolic environment in the NP.

The NP appears to be the anatomic structure in which inhaled air is integrated, and it might be a critical site that comes into contact with many inhalable pathogens, including SARS-CoV-2.^24^^,^^25^^,^^26^^,^^27^^,^^28^ Our previous data revealed the site-directed coordination of innate and adaptive antiviral immunity in the human NP in acute COVID-19 patients.^7^ However, we still lack a full understanding of localized immunological memory against SARS-CoV-2 and regulatory factors of the adaptive immunity in NP lymphoid tissue.

The compositional and predicted functional differences in the respiratory microbiome have been gaining increasing interest, and the importance of the respiratory microbiome with respect to immunity protection, is now generally recognized.^29^^,^^30^^,^^31^^,^^32^ Determining the microbial composition and its potential to cause adverse changes in the upper airway mucosa is essential for developing new ways to restrict respiratory viral infections in human. We already determined that the presence of certain components in the nasal microbiome strengthens the frontline antiviral immune response in the epithelium by modulating innate immune mechanisms.^12^^,^^33^ We assume that commensal bacteria would be distributed in the NP mucosa and there could be interactions between the commensal microbiome and immune responses in the NP, potentially allowing the NP microbiome to boost its interactions with antiviral immune responses including adaptive immune cells.

Our data revealed that *Streptococcus*, *Staphylococcus*, *Haemophilus*, and *Cutibacterium* were the most abundant genera in healthy human NP mucosa, and *S*. *aureus* complex, *H*. *influenzae*, *S*. *epidermidis*, *C*. *acnes*, and *S*. *intermedius* were cornerstone commensal species. Interestingly, we identified *S*. *aureus* complex abundance as the primary discriminating factor among NP microbial communities. Although members of the *S*. *aureus* complex are known to share pathogenic traits such as beta-hemolysis and coagulase activity,^19^^,^^34^^,^^35^ the current findings suggest that, in the NP environment, these bacteria may function as physiological commensals. A methodological limitation of our study is the restricted species-level resolution of 16S rRNA data, particularly for closely related taxa such as the *S*. *aureus* complex. This limitation was unavoidable because nasal swab samples are extremely low in microbial biomass; despite deep shotgun sequencing, host DNA dominated, leaving too few microbial reads for reliable community-wide analysis (Table S4). This challenge is well recognized in upper-airway microbiome research and made full-length 16S rRNA sequencing the most practical option in our setting.^36^^,^^37^ To substantiate species-level assignment, we performed shotgun metagenomic analysis on selected samples. Although microbial read recovery was limited, direct mapping confirmed that *S*. *aureus* was the predominant species in samples enriched for *S*. *aureus* complex (Figure S1), reinforcing its likely role in shaping localized immune responses in the NP. Further studies employing culture-based methods or host DNA depletion strategies are warranted to determine the contribution of individual *S*. *aureus* complex members and their functional interactions within the NP mucosa.

Our results show that the distribution of *S*. *aureus* complex can correlate with the frequencies of memory T and B cells. Our data reveal that the frequencies of CD4^+^ T_RM_, CD8^+^ T_RM_, GC B, and B_RM_ cells were high in the NPs of healthy donors with a high prevalence of *S*. *aureus* complex, and the population of plasmablasts in the NP was completely reduced. In particular, the prevalence of *S*. *aureus* complex in the NP was highest among BR donors, and the frequencies of CD4^+^ T_RM_, CD8^+^ T_RM_, and B_RM_, correlated significantly with a high distribution of *S*. *aureus* complex in those donors. As expected, the frequency of plasmablasts correlated inversely with abundance of *S*. *aureus* complex in the NPs of BR donors. The frequencies of tissue-resident memory cells and humoral immune responses in the lymphoid tissue of the upper airway appeared to be altered depending on the presence of *S*. *aureus* complex. Although we did not verify a potential mechanism by which COVID-19 vaccination and SARS-CoV-2 infection might change the microbiome communities, our results demonstrate crosstalk between the abundance of a particular commensal bacterium and the frequencies of tissue-resident memory T or B cells in the upper airway where SARS-CoV-2 infection begins.

While certain signals such as TGF-β, IL-15, and retinoic acid are known to promote tissue residency programs in lymphocytes,^38^^,^^39^ the specific factors that maintain tissue-resident memory cells in the unique microenvironment of the NP remain poorly understood. Our findings suggest that commensal bacteria play a crucial role in programming NP tissue-resident memory cells through distinct metabolic pathways that intersect with tissue residency signals. Analysis of *S*. *aureus* complex-high samples revealed enrichment in two key pathways that could influence the survival of T cells and tissue-resident lymphocyte maintenance. First, enrichment of the mevalonate pathway I and carotenoid biosynthesis points to a mechanism whereby bacterial carotenoids could serve as precursors for local retinoid production. This finding is particularly significant as retinoic acid has recently emerged as a fundamental coordinator of tissue immunity, driving tissue residency programs across multiple organs and synergizing with TGF-β to establish and maintain tissue-resident lymphocytes in mucosal tissues.^40^^,^^41^ Second, enhanced nitrogen metabolism pathways, including arginine metabolism and urea cycle, suggest a complex metabolic crosstalk where *S*. *aureus* complex may create favorable conditions for the activation or survival of memory cells through secondary metabolites such as ornithine and proline or adaptive responses to metabolic stress.^20^^,^^42^ Given that arginine metabolism directly shapes T cell metabolic fitness, these microbial pathways could also interact with immune checkpoint signaling pathways (e.g., PD-1 and CTLA-4) that restrain T cell activity, thereby potentially influencing the balance of survival and exhaustion in NP-resident lymphocytes.^43^ These potential immunometabolic interactions highlight a distinct dimension of host-microbe crosstalk that warrants further functional investigation.

Notably, previous studies have demonstrated that *S*. *aureus* infection can induce T_RM_ cell responses in barrier tissues such as the murine lung and human skin explants.^44^^,^^45^ To our knowledge, our study is the first to quantitatively link *S*. *aureus* colonization levels with T_RM_ and B_RM_ frequencies in a human airway mucosa. This represents an underexplored dimension of host-microbiota interaction, where commensal *S*. *aureus* acts not merely as a bystander or pathogen, but as a potential local immune modulator shaping tissue-resident lymphocyte landscapes in the nasopharynx. Unlike prior experimental models, which largely involved induced immune activation, our human data reflect steady-state colonization, suggesting that the immunological imprinting by *S*. *aureus* may emerge even in the absence of overt inflammation. Interestingly, this stands in contrast to murine nasal mucosa, where natural *S*. *aureus* colonization fails to induce significant T_RM_ responses.^44^ These findings highlight the NP niche as an immune environment where *S*. *aureus* colonization is specifically associated with the presence of tissue-resident memory lymphocytes, suggesting a unique interaction between this commensal and the NP mucosal immune system.

Although there were no differences depending on the COVID-19 vaccine and infectivity, our data indicate the abundant mucosal microbiome predominantly distributed in NP. We also suggest that the individual enhanced abundance of *S*. *aureus* complex supports the maintenance of tissue-resident memory cells and induction of GC responses in the NP. Importantly, this represents the first evidence of a commensal-driven association between *S*. *aureus* colonization and mucosal immune memory in a natural human tissue. These findings offer new insights into host-microbiome interactions that could help to uncover the microbial environment for localized immunological memory in upper airway.

### Limitations of the study

Our study was specially designed to determine localized host-microbiome interaction in the NP lymphoid tissues but there are limitations to address in future research. We do not have longitudinal samples to precisely map the duration of immunological changes, and could not identify antigen-specific T or B cells in NP lymphoid tissue by peptide stimulation. In addition, the number of donors who underwent simultaneous brushing for flow cytometry and swabs for microbiome analysis from the NP was rather small, especially HD and CV donors. Furthermore, it was difficult to obtain samples from control donors who had no exposure to COVID-19 and had not been vaccinated, considering the timing of the COVID-19 pandemic in Korea, which prevented sufficient comparison between groups. These observed distribution patterns warrant further investigation into potential relationships between NP bacterial populations and COVID-19 infection or vaccination status in larger cohorts. Our microbial pathway analysis is based on predictive inferences from 16S rRNA sequencing data. Accordingly, hypotheses regarding immunometabolic pathways remain speculative and require validation through metabolomic profiling, microbial functional assays, and functional immunological assays.

## Resource availability

### Lead contact

Further information and requests for resources and reagents should be directed to and will be fulfilled by the lead contact, Hyun Jik Kim (hyunjerry@snu.ac.kr).

### Materials availability

The study did not generate any materials.

### Data and code availability

• The raw sequence data for each sample were deposited in the NCBI Sequence Read Archive. Accession numbers are listed in the key resources table.
• This study does not report original code.
• Any additional information required to reanalyze the data reported in this article is available from the lead contact upon request.

## Acknowledgments

This work was supported by the Basic Science Research Program through the 10.13039/501100003725National Research Foundation of Korea, funded by the Ministry of Education (2022R1A2C2011867) awarded to H.J.K and by the 10.13039/501100014188Ministry of Science and ICT (RS 2023-00222762), Korea awarded to H.J.K. This research was also supported by a grant from the Korean Health Technology R&D Project through the Korean Health Industry Development Institute, funded by the Ministry of Health and Welfare of the Republic of Korea (HI23C0795 awarded to H.J.K), and by the National Research Foundation of Korea (NRF), funded by the Ministry of Science and ICT (RS-2023-00209621 awarded to Y.-J.B).

## Author contributions

S.-T.P., J.W., and S.J. designed the experiments and wrote the study protocols. S.-T.P. performed the metabolite analysis and bioinformatic processing. S.K., H.S., and S.H.L. were responsible for laboratory processing of samples. S.J. ran statistical analysis. Y.-J.B. and H.J.K. wrote the manuscript and contributed to interpretation of the results. All authors approved the final manuscript.

## Declaration of interests

The authors declare that the research was conducted in the absence of any commercial or financial relationships that could be construed as potential conflicts of interest.

## STAR★Methods

### Key resources table

**Table:** 

REAGENT or RESOURCE	SOURCE	IDENTIFIER
**Antibodies**		

PE/Cy7-conjugated anti CCR7, clone G043H7	Biolegend	Cat# 353225; RRID: AB_11126145
PE/Dazzle594-conjugated anti CD103, Ber-ACT8	Biolegend	Cat# 350223; RRID: AB_2716188
APC/Cy7-conjugated anti CD19, clone HIB19	Biolegend	Cat# 302217; RRID: AB_314247
PE/Cy5-conjugated anti CD20, clone 2H7	Biolegend	Cat# 302307; RRID: AB_314256
FITC-conjugated anti CD3, clone UCHT1	Biolegend	Cat# 300452; RRID: AB_2562046
BV785-conjugated anti CD4, clone OKT4	Biolegend	Cat# 317441; RRID: AB_2563242
AF700-conjugated anti CD45, clone HI30	Biolegend	Cat# 304024; RRID: AB_493760
BV510-conjugated anti CD45RA, clone HI100	Biolegend	Cat# 304141; RRID: AB_2561947
PerCP/Cy5.5-conjugated anti CD324, clone 67A4	Biolegend	Cat# 324113; RRID: AB_2076797
BV605-conjugated anti CD326, clone 9C4	Biolegend	Cat# 324223; RRID: AB_2562518
BV650-conjugated anti CD38, clone HB-7	Biolegend	Cat# 356619; RRID: AB_2566232
BV711-conjugated anti CD69, clone FN50	Biolegend	Cat# 310943; RRID: AB_2566466
APC-conjugated anti CD8, clone SK1	Biolegend	Cat# 344722; RRID: AB_2075388
BV421-conjugated anti CXCR5, clone J252D4	Biolegend	Cat# 356920; RRID: AB_2562302
PE-conjugated anti PD-1, clone EH12.2H7	Biolegend	Cat# 329905; RRID: AB_940481

**Chemicals peptides, and recombinant proteins**		

RPMI 1640	Welgene	Cat no: LM011-01
Fetal Bovine Serum	ThermoFisher	Cat no: 16000044
GlutaMAX	ThermoFisher	Cat no: 35050061
Human BD Fc Block	BD biosciences	Cat no: 564220
Brilliant Stain Buffer Plus	BD biosciences	Cat no: 566385
RBC Lysis Buffer	Biolegend	Cat no: 420301

**Software and algorithms**		

Prism v10.6.1	GraphPad	https://www.graphpad.com/
FlowJo v10.7	BD biosciences	https://www.flowjo.com/

**Deposited data**		

Metagenome sequencing data	This paper	BioProject: PRJNA1194306

### Experimental model and study participant details

#### Study participants

Thirty-one subjects were enrolled in this study, 21 males and 10 females. Their mean age was 42.1 years, and they were all referred to the Department of Otorhinolaryngology at Seoul National University Hospital (Seoul, Korea) between January and June 2024, primarily for nasal surgery. None of the subjects showed signs of acute infection, and all showed negative results in the allergy test (Multiple allergens simultaneous test, MAST). Subjects who had taken any kind of antibiotics within the preceding 2 months; were pregnant or smokers; had diseases or medication histories related to asthma; or had any other chronic diseases were excluded. This study was performed according to the Helsinki Declaration and was approved by the Institutional Review Board of Seoul National University College of Medicine, Seoul, Korea (IRB No. 2305-149-1434). Written informed consent was obtained from all participants before sample collection. The subjects were classified into 4 groups according to their SARS-CoV-2 infection and COVID-19 vaccination history: healthy vaccinated (HV) donors, breakthrough (BR) donors, hybrid immunity (HD) donors, and convalescent (CV) donors (Figure 1A). All subjects included in this study had received at least one dose of COVID-19 vaccines, and those with a history of SARS-CoV-2 infection were confirmed by SARS-CoV-2 polymerase chain reaction during the omicron era in Korea.

### Method details

#### Sample collection and processing

NPs were accurately observed using an intranasal endoscope and both the NP swabbing (for microbial analysis) and brushing (for flow cytometry) were performed by an otorhinolaryngologist (HJ Kim) (Video S1). A flocked brush (Vansco, Seoul, Korea) was gently inserted into one nostril along the floor of the nasal cavity into the posterior NP. Mucus from the NP mucosa was collected individually from 31 subjects using sterile 3M Quick swabs (3M Microbiology Products, ST Paul, MN, USA). Each swab and brush sample placed in an individual tube containing RPMI 1640 (Welgene, Gyeongsangbuk-Do, Korea) supplemented with 10% fetal bovine serum (ThermoFisher, Waltham, MA, USA). The swabs with mucus were fixed in a fixative solution and transported immediately to the laboratory for identification and microbial analysis (full-length 16S rRNA sequencing on a PacBio Sequel IIe system). After collection, the brushing samples were vortexed briefly within the capped collection tube to release the cells for flow cytometry. Additional medium was then used to rinse adherent cells from the brush into a 40 μm mesh filter over a 50 mL conical tube. The filtered cell suspension was centrifuged at 500 x g for 7 min at 4°C, and then the brushed cells were processed for downstream applications.

Video S1. NP swabbing and brushing

#### Flow cytometry

Fresh NP cells were isolated using Ficoll-Paque PLUS (GE Healthcare, Chicago, Il, USA) and stained for surface and intracellular markers. The antibodies and clones used are described in key resources table (Biolegend. San Diego, CA, USA). Briefly, the cells were washed with fluorescence-activated cell sorting (FACS) buffer (PBS with 2% heat-inactivated fetal bovine serum) and then resuspended with a surface staining antibody cocktail for 20 min at room temperature. The surface-stained cells were fixed for 30 min at room temperature in fixation buffer, washed with permeabilization buffer (Tonbo (Cytek Biosciences), Fremont, CA, USA. catalog no. TNB-0607-KIT), and washed again with FACS buffer. Flow cytometry data were collected using a five-laser Cytek Aurora flow cytometer (Cytek Biosciences) and analyzed using FlowJo V 10.7 software (Figure S7).

#### DNA extraction

DNA was extracted using a DNeasy PowerSoil Pro Kit (Qiagen, Hilden, Germany) according to the manufacturer's instructions. The extracted DNA was quantified using VICTOR Nivo™ (Perkin Elmer) with PicoGreen reagents. The extracted DNA was dissolved in sterile water containing 40 μg/mL RNase A and was quantified with a Nano Quant Infinite M200 spectrophotometer (Tecan, Männedorf, Switzerland) as the ratio of absorbance values at 260 and 280 nm (A_260_/A_280_). To monitor possible reagent- or kit-derived contamination, negative controls (buffer-only blanks) were prepared and subjected to the same extraction procedures in parallel with study samples. These blanks were evaluated by TapeStation during the library QC step; no amplifiable DNA was detected, and therefore no sequencing libraries were generated.

#### Metagenome amplicon sequencing and taxonomic analysis

Subsequent metagenome amplicon sequencing and initial data processing were performed by Macrogen (Seoul, Republic of Korea). Full-length 16S rRNA gene amplicon libraries (V1–V9 regions amplified with 27F/1492R primers) were prepared using a PacBio 16S full-length library kit 3.0. Sequencing was conducted on a PacBio Sequel IIe system (Table S1). The raw sequencing data were processed as follows. Adapter and primer sequences were removed using Cutadapt v3.2.^46^ Subsequently, reads were demultiplexed, denoised, and merged using DADA2 v1.18.0.^47^ During this process, low-quality reads were trimmed and filtered, and chimeric sequences were removed. A total of 2,098,013 HiFi reads (67,678 reads on average) from 31 samples was recovered (Table S2). The resulting amplicon sequence variants (ASVs) were aligned to reference organisms using BLAST+ (v2.9.0+).^48^ When multiple species showed the same top-hit, species were assigned if the similarity score of the next closest species was ≥0.2% lower than that of the top-hit species. If the difference was <0.2%, those species were also included in the assignment. The raw sequence data for each sample were deposited in the NCBI Sequence Read Archive.

#### Metagenome shotgun sequencing and taxonomic analysis

HV4, BR1, BR2, BR17, and BR18 was analyzed by shotgun metagenome sequencing. The sequencing library was prepared using TruSeq Nano DNA Library Prep Kit. Sequencing was conducted on a NovaSeq X with 150 bp paired-end reads (Table S3). The raw data were processed using Kneaddata (v0.10.0)^49^ to remove host-derived reads with human genome reference hg37dec_v0.1 (Table S4). Host-filtered reads were then aligned to type strain genomes of *Staphylococcus aureus* complex members—*S. aureus* (GCF_000013425.1), *S. roterodami* (GCF_904830535.1), *S. argenteus* (GCF_000236925.1), and *S. schweitzeri* (GCF_900636685.1) using Bowtie2 (v2.2.3).^50^ The reference genome indices were built using Bowtie2-build (v2.4.5). Mapping results were processed and summarized using Samtools (v1.9). The raw sequence data for each sample were deposited in the NCBI Sequence Read Archive.

#### Microbiome diversity analysis and functional prediction

Microbial diversity was assessed using the phyloseq package in R (v1.48.0).^51^ Alpha diversity was analyzed by measuring Shannon and Gini-Simpson indexes and beta diversity was analyzed via Bray-Curtis dissimilarity and Principal Coordinate Analysis (PCoA). PERMANOVA evaluated group differences in beta diversity, and group-specific taxa were identified with ANCOM-BCII (Analysis of Composition of Microbiomes with Bias Correction 2) and random forest analyses using randomForest package (version 4.7-1.2) in R.^52^^,^^53^ For Random Forest, species-level relative abundance data were used as input, and the model was trained with 1000 trees (ntree = 1000) using default parameters, and feature importance was assessed using the Mean Decrease Gini index. Features identified as discriminative by both ANCOM-BC II and Random Forest were further evaluated by Wilcoxon rank sum tests. Predicted microbial function was inferred using PICRUSt2 (v2.5.3)^54^ and mapped to MetaCyc pathways, with differential abundances analyzed using DESeq2.^55^ Pathway visualizations were created using the ggpicrust2 package (v1.7.3).^56^

### Quantification and statistical analysis

Due to the small and unequal sample sizes across experimental groups, non-parametric tests were used for all comparisons of continuous variables to ensure statistical robustness. Specifically, comparisons between two independent groups were performed using the Wilcoxon rank sum test. For comparisons among three groups, the Kruskal-Wallis test followed by Dunn’s multiple-comparison test was used. Spearman’s rank order correlation was used for nonparametric correlation analyses. To evaluate linear relationships, univariate simple linear regression was first applied. To account for potential confounding by age and sex, we then fit multivariable linear regression models. All regression models used heteroscedasticity-consistent (HC3) standard errors to address potential heteroscedasticity. All p values are from two-sided comparisons. P values for multiple comparisons were corrected using the Benjamini-Hochberg FDR method. Descriptive statistics from the compiled flow cytometry data and statistical analyses were performed using Prism (GraphPad). Graphs were also generated using Prism (GraphPad) and the R Studio corrplot package and ggplot2 package (v.3.5.1). Statistical significance was defined as *p*-values of ≤ 0.05 (∗), *p* ≤ 0.01 (∗∗), *p* ≤ 0.001 (∗∗∗) and *p* ≤ 0.0001 (∗∗∗∗). Statistical details can be found in the figure legends.
