# Supplementary material for: Distinct commensal bacteria in human nasopharyngeal lymphoid tissue associated with localized immunological memory
Source: iScience. 2025 Dec 30;29(2):114579. doi: 10.1016/j.isci.2025.114579 (PMC12830300; doi:10.1016/j.isci.2025.114579)
Supplement: Document S1. Figures S1–S9 and Tables S1–S4 [file mmc1.pdf]

iScience, Volume *29*

Supplemental information

Distinct commensal bacteria in human nasopharyngeal lymphoid tissue associated with localized immunological memory

Seung-Taek Park, Jina Won, Siyeon Jin, Sujin Kim, Haeun Shin, Su Hyun Lim, Ye-Ji Bang, and Hyun Jik Kim

Supplementary information

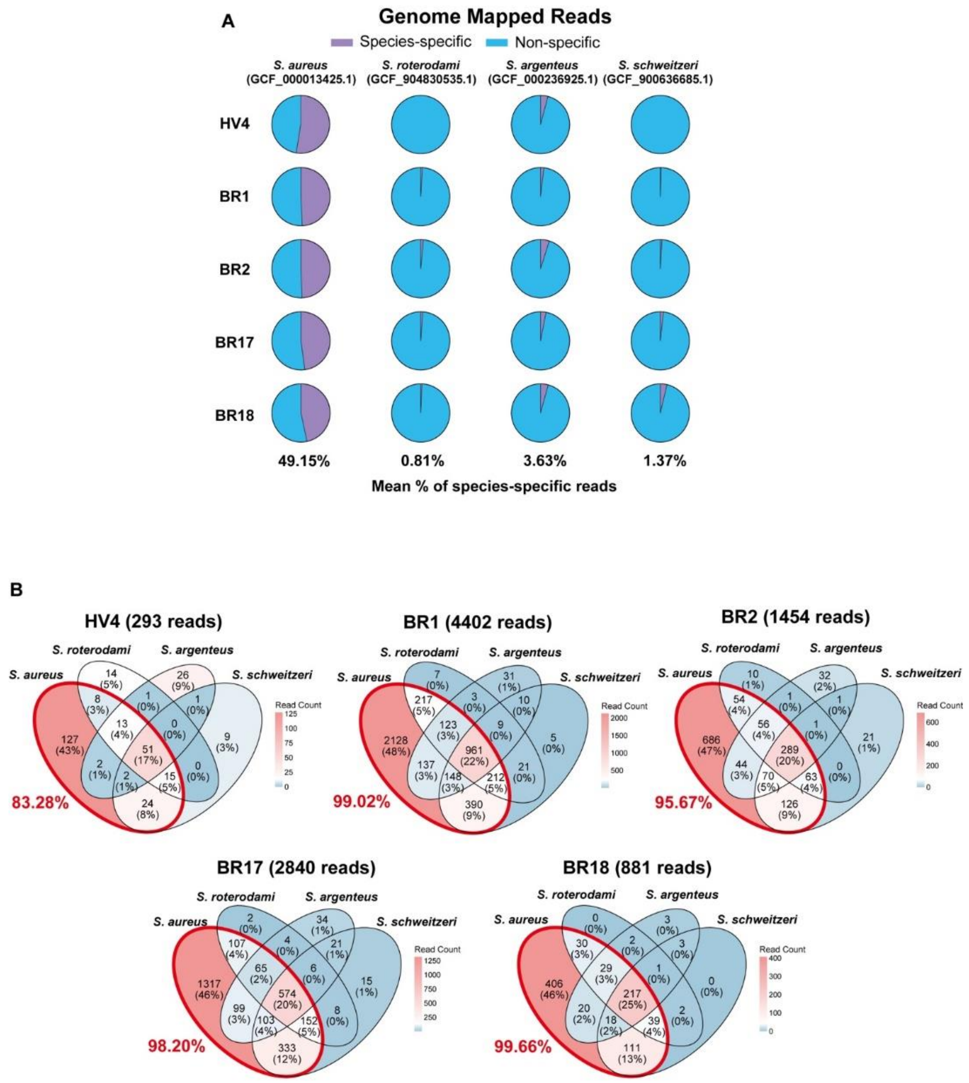

Figure S1. Shotgun metagenomic read mapping to Staphylococcus aureus complex reference genomes, related to Figure 1.

a, Pie charts show the proportion of species-specific (purple) versus non-species-specific (blue) reads mapped to each *S. aureus* complex genome from five NP samples (HV4, BR1, BR2, BR17, BR18). Host-filtered shotgun metagenomic reads were aligned to four reference genomes: *S. aureus* (GCF_000013425.1), *S. roterodami* (GCF_904830535.1), *S. argenteus* (GCF_000236925.1), and *S.*

schweitzeri (GCF_900636685.1). Each chart represents the subset of reads that mapped to that species' genome. **b,** Venn diagrams illustrate the overlap in mapped reads across the four *S. aureus* complex genomes for each sample. Numbers indicate the count of reads mapping uniquely or multiply to each combination of species. Color shading reflects relative read counts (scale at bottom right). Red percentages represent the fraction of reads mapped to *S. aureus* among those mapped to the S. aureus complex.

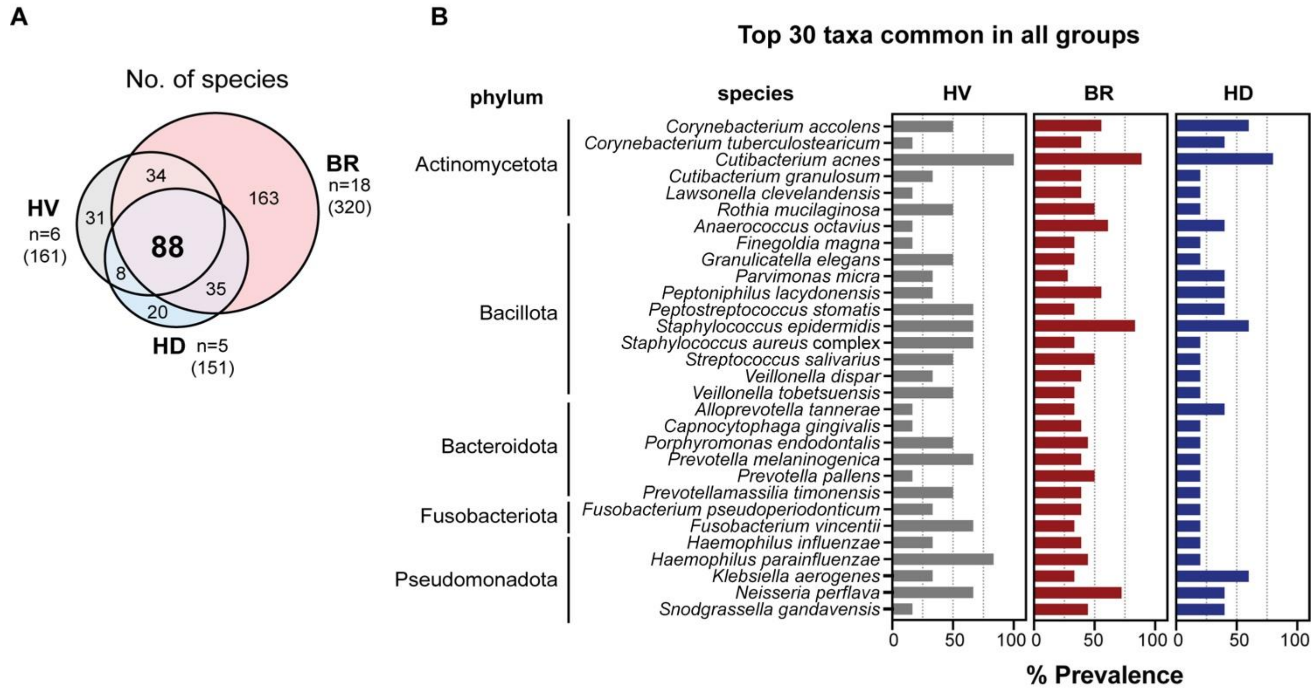

Figure S2. Distribution of shared bacterial species among study groups, related Figure 2.

a, Venn diagram showing the number of species shared across the Healthy Vaccinated (HV), Breakthrough (BR), and Hybrid Immunity (HD) groups. A total of 88 species were common to all three groups and defined as the core nasopharyngeal microbiome. **b,** Bar plots showing the prevalence (%) of the top 30 most common species among the 88 core taxa, organized by phylum. Species were selected based on the highest mean prevalence across all samples. Bars are color-coded by group (HV: gray, BR: red, HD: blue). The shared species include multiple representatives of *Staphylococcus, Streptococcus, Veillonella, Prevotella,* and *Haemophilus*, reflecting the conserved microbial structure of the human nasopharynx across different COVID-19 exposure histories.,

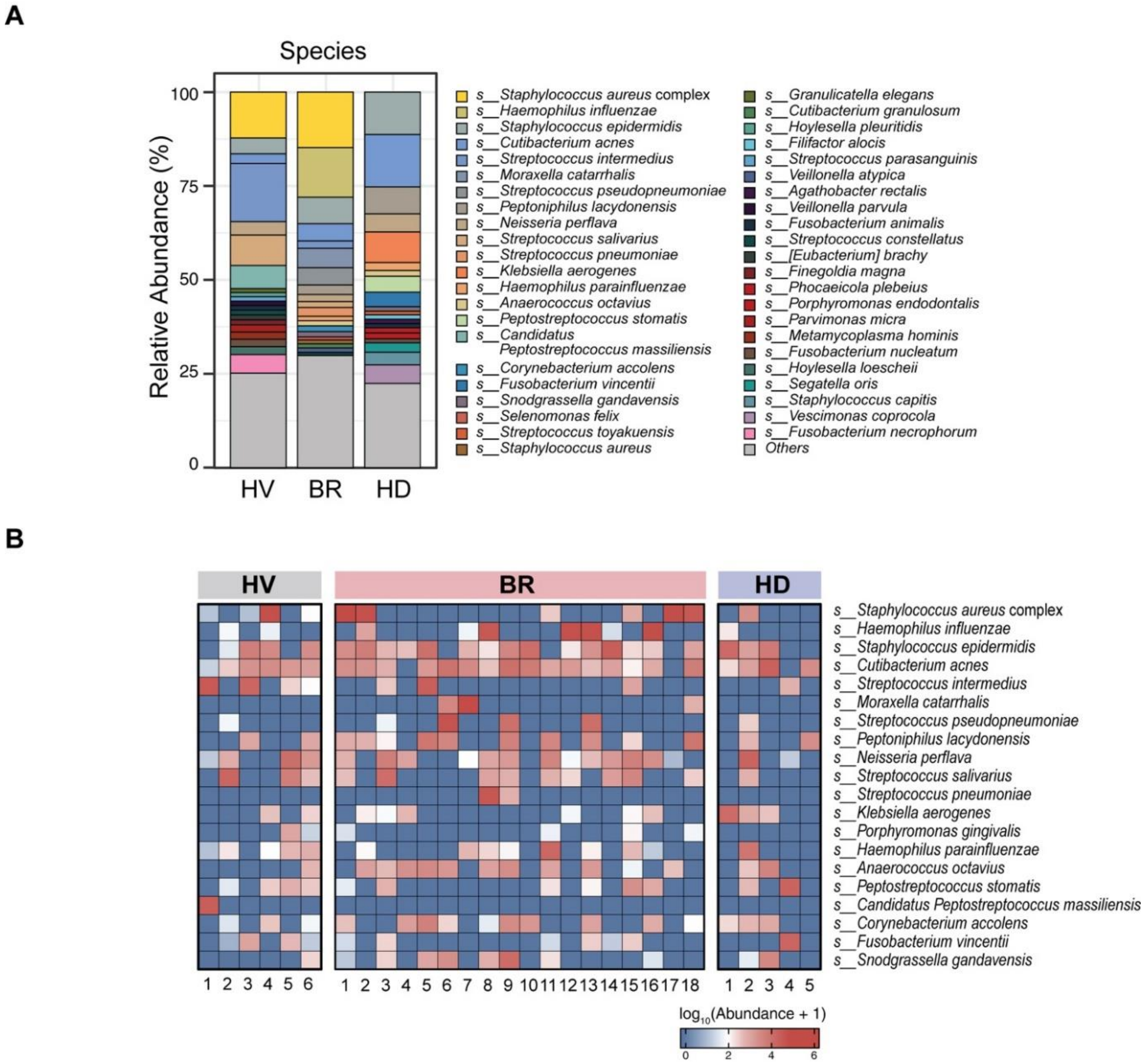

Figure S3. Species-level composition analysis among HV, BR, and HD groups, related Figure 2.

a, Stacked bar showing the mean relative abundance of bacterial species in each group (HV, BR, HD). Colors represent different species, with the legend provided to the right. **b,** Heatmap displaying the log10-transformed abundance of key bacterial species across individual samples in each group. Columns represent samples, rows represent species, and color intensity indicates abundance (blue: low, red: high).

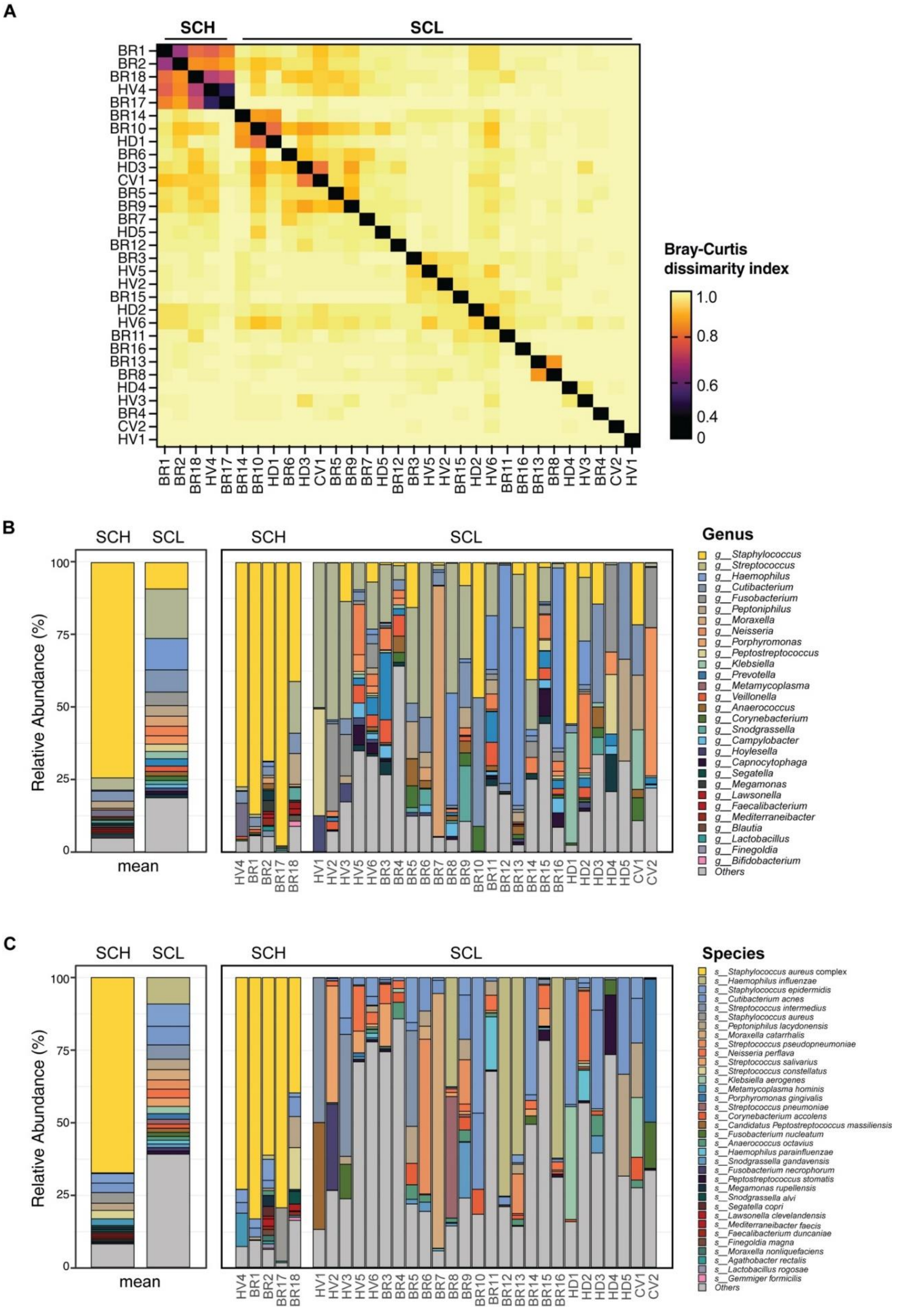
A
SCH
SCL
BR1 BR2 BR18 HV4 BR17 BR14 BR10 HD1 BR6 HD3 CV1 BR5 BR9 BR7 HD5 BR12 BR3 HV5 HV2 BR15 HD2 HV6 BR11 BR16 BR13 BR8 HD4 HV3 BR4 CV2 HV1
Bray-Curtis
dissimarity index
1.0
0.8
0.6
0.4
0
B
SCH
SCL
SCH
SCL
100
75
50
25
0
Relative Abundance (%)
mean
HV4 BR1 BR2 BR17 BR18 HV1 HV2 HV3 HV5 HV6 BR3 BR4 BR5 BR6 BR7 BR8 BR9 BR10 BR11 BR12 BR13 BR14 BR15 BR16 HD1 HD2 HD3 HD4 HD5 CV1 CV2
Genus
g__Staphylococcus
g__Streptococcus
g__Haemophilus
g__Cutibacterium
g__Fusobacterium
g__Peptoniphilus
g__Moraxella
g__Neisseria
g__Porphyromonas
g__Peptostreptococcus
g__Klebsiella
g__Prevotella
g__Metamycoplasma
g__Veillonella
g__Anaerococcus
g__Corynebacterium
g__Snodgrassella
g__Campylobacter
g__Hoylesella
g__Capnocytophaga
g__Segatella
g__Megamonas
g__Lawsonella
g__Faecalibacterium
g__Mediterraneibacter
g__Blautia
g__Lactobacillus
g__Finegoldia
g__Bifidobacterium
Others
C
SCH
SCL
SCH
SCL
100
75
50
25
0
Relative Abundance (%)
mean
HV4 BR1 BR2 BR17 BR18 HV1 HV2 HV3 HV5 HV6 BR3 BR4 BR5 BR6 BR7 BR8 BR9 BR10 BR11 BR12 BR13 BR14 BR15 BR16 HD1 HD2 HD3 HD4 HD5 CV1 CV2
Species
s__Staphylococcus aureus complex
s__Haemophilus influenzae
s__Staphylococcus epidermidis
s__Cutibacterium acnes
s__Streptococcus intermedius
s__Staphylococcus aureus
s__Peptoniphilus lacydonensis
s__Moraxella catarrhalis
s__Streptococcus pseudopneumoniae
s__Neisseria perflava
s__Streptococcus salivarius
s__Streptococcus constellatus
s__Klebsiella aerogenes
s__Metamycoplasma hominis
s__Porphyromonas gingivalis
s__Streptococcus pneumoniae
s__Corynebacterium accolens
s__Candidatus Peptostreptococcus massiliensis
s__Fusobacterium nucleatum
s__Anaerococcus octavius
s__Haemophilus parainfluenzae
s__Snodgrassella gandavensis
s__Fusobacterium necrophorum
s__Peptostreptococcus stomatis
s__Megamonas rupellensis
s__Snodgrassella alvi
s__Segatella copri
s__Lawsonella clevelandensis
s__Mediterraneibacter faecis
s__Faecalibacterium duncaniae
s__Finegoldia magna
s__Moraxella nonliquefaciens
s__Agathobacter rectalis
s__Lactobacillus rogosae
s__Gemmiger formicilis
Others

Figure S4. Detailed analysis of microbial composition in SCH and SCL groups, related Figure 3.

a, Heatmap showing Bray-Curtis dissimilarity matrix between all samples. Color scale indicates dissimilarity index (0: identical, 1.0: completely different). **b,** Relative abundance of bacterial genera. Left: mean abundance in SCH and SCL groups. Right: individual sample profiles. Colors represent different genera according to legend. **c,** Relative abundance of bacterial species. Left: mean abundance in SCH and SCL groups. Right: individual sample profiles. Colors represent different species according to legend.

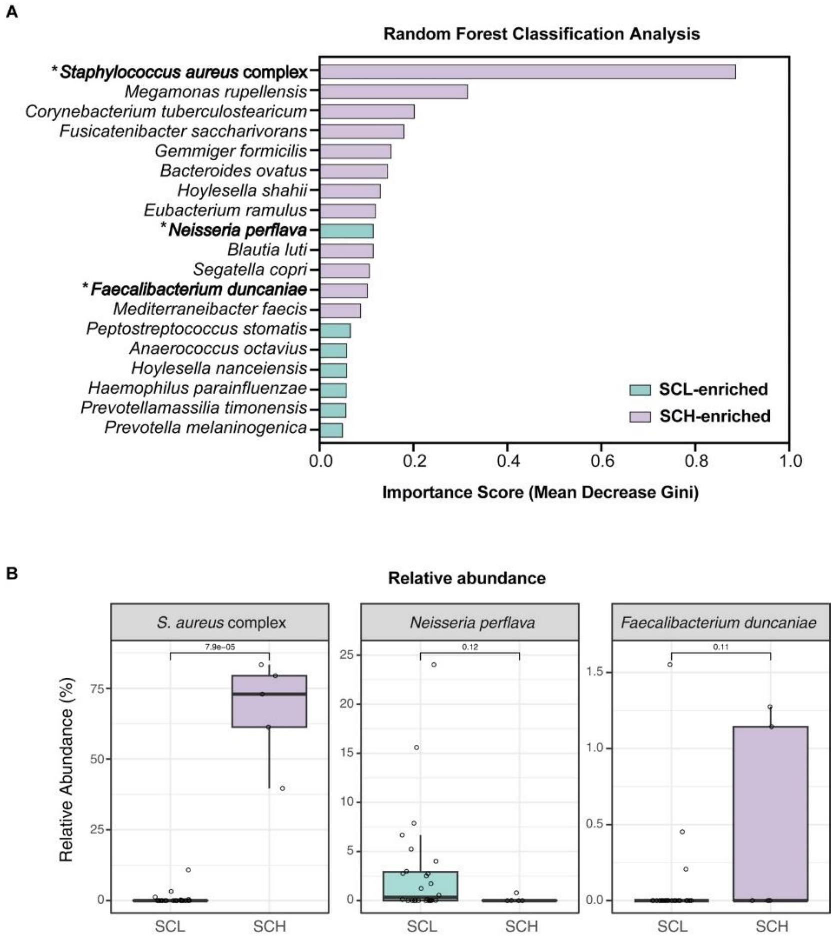

Figure S5. Random forest classification and validation of differentially abundant species in SCH and SCL groups, related to Figure 3.

a, Random forest classification was performed using species-level relative abundance profiles to distinguish NP microbiota of S. aureus complex-high (SCH) and -low (SCL) groups. The top-ranking 20 features by importance score (Mean Decrease Gini) are shown. Bar color indicates the group in which each species was enriched (purple: SCH, teal: SCL), based on log2 fold-change direction. **b,** Box plots showing relative abundance of three overlapping species identified by both ANCOM-BC II and random forest analysis. Each dot represents an individual sample with median line. P-values from Wilcoxon rank sum test are shown.

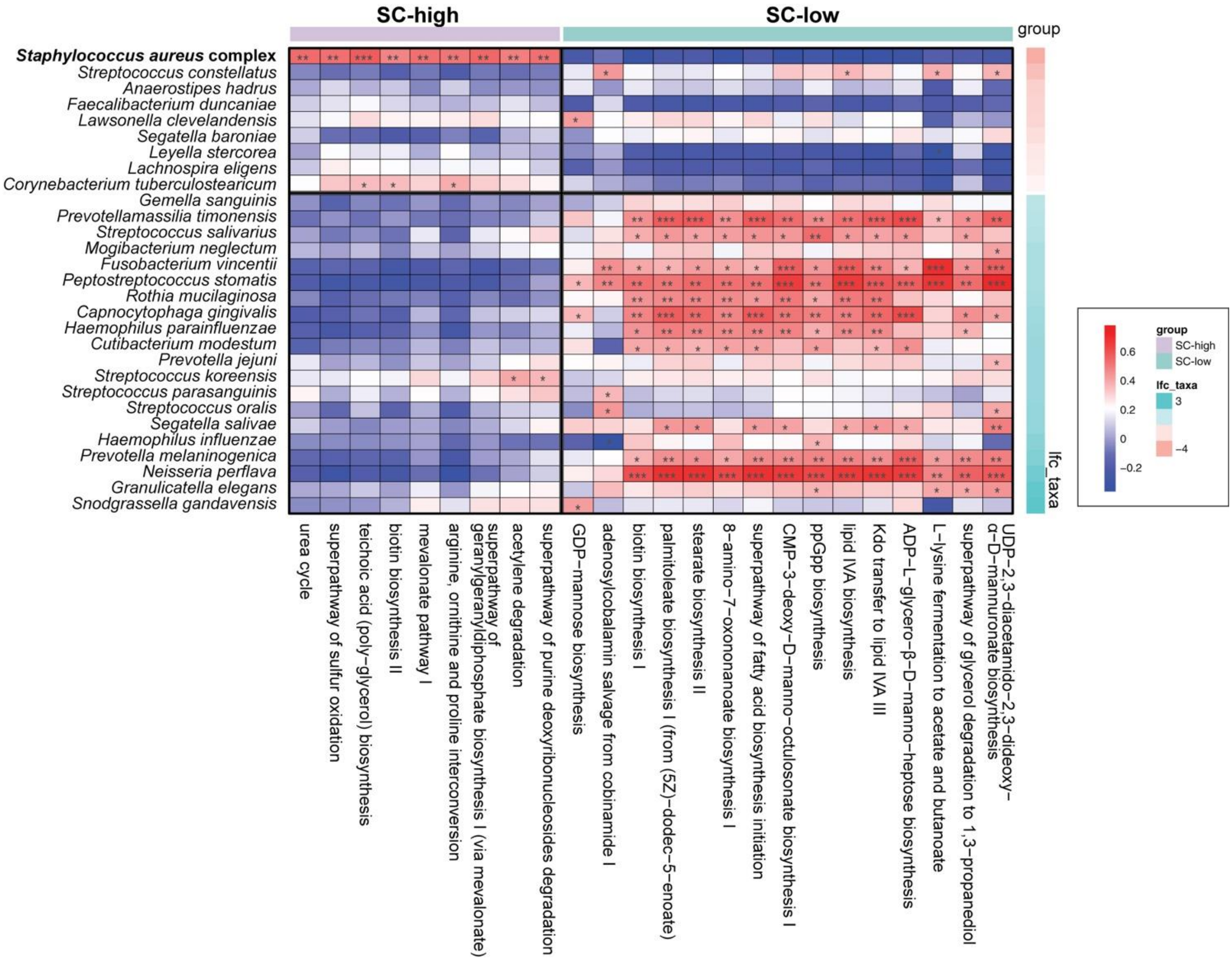

Figure S6. Correlation between bacterial species and predicted metabolic pathways, related to Figure 3.

Heatmap showing correlations between bacterial species (rows) and predicted MetaCyc pathways (columns) enriched across SC-high and SC-low groups. Color scale indicates correlation strength (red: positive, blue: negative correlation). Right side color bar indicates group enrichment (pink: SC-high, blue: SC-low). Pearson correlation analysis with FDR adjusted p value is shown: *p<0.05, **p<0.01, ***p<0.001.

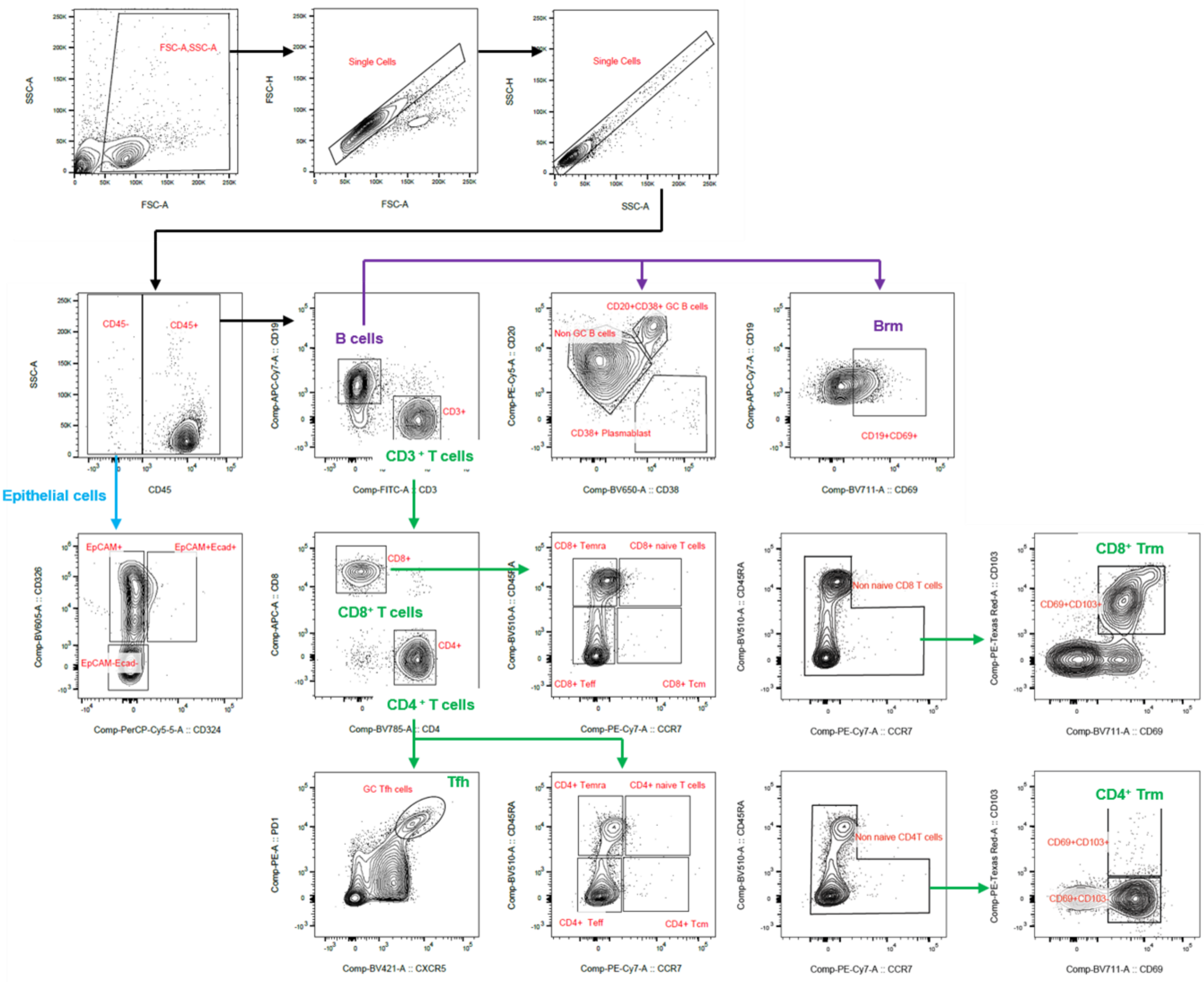

Figure S7. Flow cytometry gating strategy for immune cell populations in nasopharyngeal lymphoid tissue, related to Figure 4.

Representative flow cytometry plots showing sequential gating strategy for analysis of immune cell populations. The gating strategy identifies epithelial cells, CD4$^+$ T cells, CD8$^+$ T cells, CD19$^+$ B cells, CD4$^+$ T$_{RM}$ (CD69$^+$CD103$^+$ or CD69$^+$CD103$^-$), CD8$^+$ T$_{RM}$ (CD69$^+$CD103$^+$), Tfh (CXCR5$^+$PD1$^+$), and B$_{RM}$ (CD19$^+$CD69$^+$) from nasopharyngeal lymphoid tissue samples.

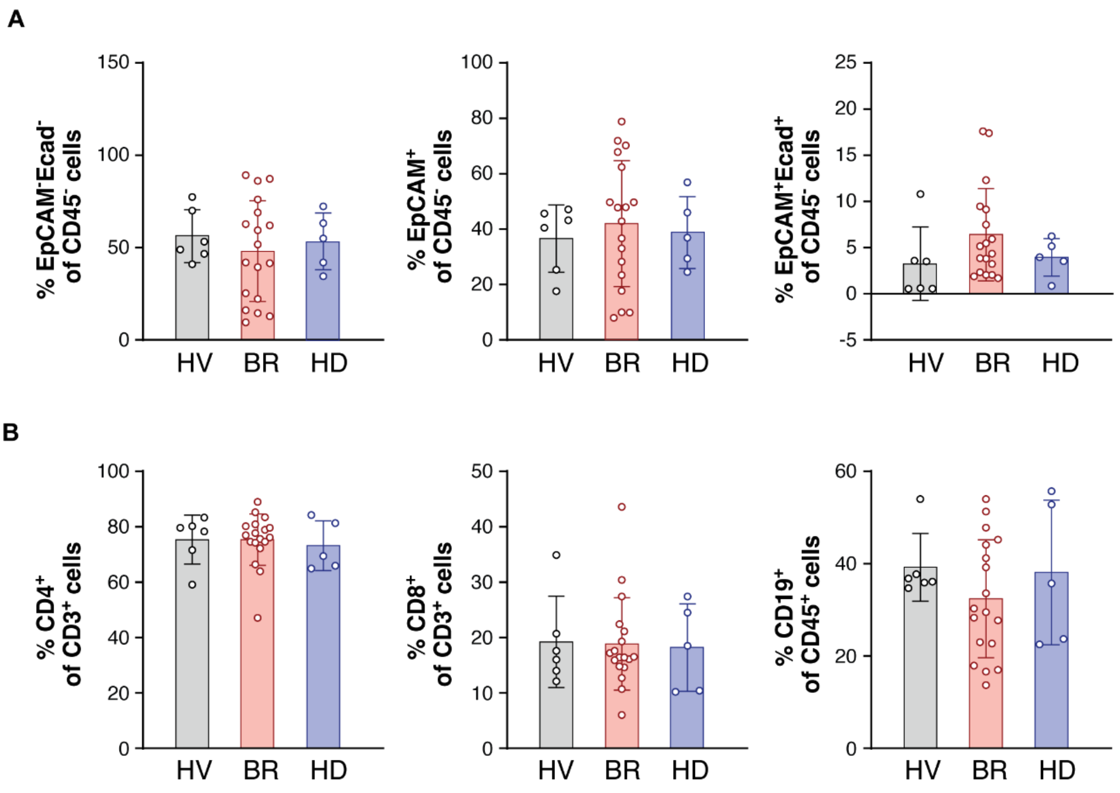

Figure S8. Analysis of epithelial cells and lymphocyte populations in nasopharyngeal lymphoid tissue across study groups, related to Figure 4.

a, Frequency of epithelial cell populations (EpCAM$^-$Ecad$^-$, EpCAM$^+$, EpCAM$^+$Ecad$^+$) among CD45$^-$ cells in HV, BR, and HD groups. **b,** Frequency of lymphocyte populations (CD4$^+$ T cells, CD8$^+$ T cells, CD19$^+$ B cells) among CD45$^+$ cells in HV, BR, and HD groups. Bars show mean ± standard deviation, with individual data points overlaid. HV: Healthy vaccinated, BR: Breakthrough, HD: Hybrid immunity.

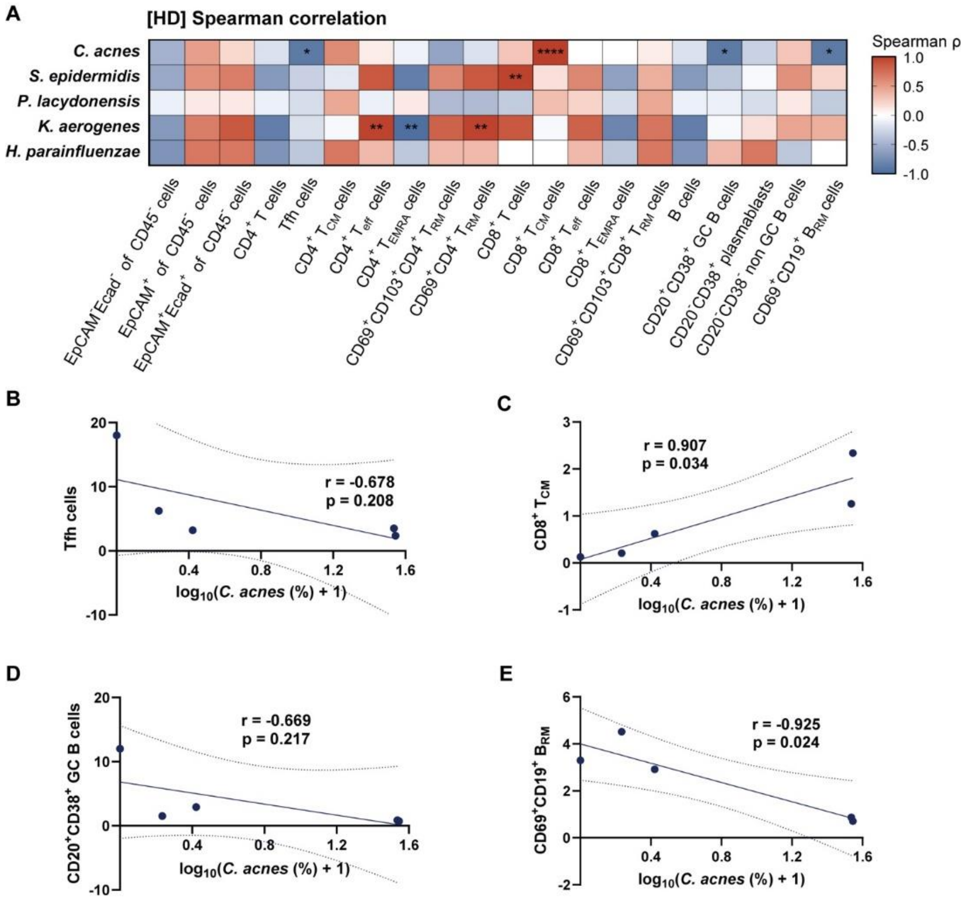

Figure S9. Correlation analysis between bacterial species and immune cell populations in HD donors, related to Figure 5.

a, Heatmap showing correlations between key bacterial species and immune cell populations in nasopharyngeal samples of HD donors (n=5). Color scale indicates correlation strength (red: positive, blue: negative correlation). Asterisks indicate statistical significance (**p* < 0.05, ***p* < 0.01, *****p* < 0.0001) determined by the Spearman correlation test. **b-e,** Scatterplots showing relationships between *C. acnes* abundance and **b,** Tfh cells, **c,** $CD8^+$ T_{CM} cells, **d,** GC B cells, and **e,** $CD69^+CD19^+$ B_{RM} cells in HD donors. Pearson correlation coefficients (*r*) and *p*-values are shown. Linear regression lines with 95% confidence intervals are overlaid.

Table S1. Raw data statistics of PacBio 16S full-length sequencing, related to STAR Methods.

Sample ID	HiFi Read Bases	HiFi Reads	HiFi N50	Average Read Length	Average Read quality	Average Pass
HV1	133,940,174	89,126	1,495	1,502	Q30	25
HV2	98,090,265	65,461	1,508	1,498	Q29	25
HV3	77,715,939	51,331	1,512	1,514	Q31	25
HV4	142,984,204	95,068	1,513	1,504	Q31	25
HV5	142,749,702	95,265	1,495	1,498	Q31	25
HV6	90,226,483	60,163	1,500	1,499	Q30	25
BR1	162,122,468	107,265	1,513	1,511	Q30	25
BR2	152,392,972	101,052	1,513	1,508	Q30	25
BR3	164,709,473	109,667	1,499	1,501	Q30	25
BR4	81,248,319	54,326	1,491	1,495	Q30	25
BR5	126,676,783	84,298	1,509	1,502	Q30	25
BR6	153,256,503	102,283	1,506	1,498	Q31	25
BR7	136,622,412	91,626	1,491	1,491	Q31	25
BR8	160,059,695	106,073	1,505	1,508	Q31	25
BR9	133,816,021	89,277	1,502	1,498	Q30	25
BR10	45,904,555	30,367	1,490	1,511	Q30	25
BR11	158,599,861	105,635	1,501	1,501	Q31	25
BR12	126,855,202	84,603	1,501	1,499	Q30	25
BR13	160,168,767	106,983	1,501	1,497	Q30	25
BR14	117,699,955	78,143	1,509	1,506	Q30	25
BR15	136,001,326	90,651	1,494	1,500	Q30	25
BR16	173,681,450	115,903	1,501	1,498	Q31	25
BR17	149,810,359	98,972	1,513	1,513	Q31	25
BR18	157,324,313	104,551	1,512	1,504	Q31	25
HD1	64,205,753	42,601	1,511	1,507	Q30	25
HD2	147,521,978	98,123	1,502	1,503	Q30	25
HD3	120,802,498	80,892	1,490	1,493	Q30	25
HD4	188,829,402	126,330	1,493	1,494	Q30	25
HD5	17,483,772	11,696	1,486	1,494	Q30	25
CV1	184,552,802	123,432	1,488	1,495	Q30	25
CV2	133,499,833	89,283	1,492	1,495	Q30	25

Table S2. QC statistics of PacBio 16S full-length sequencing, related to STAR Methods.

Sample Name	Raw data	Adapter & Primer Trimming	Quality Filter	QC Remain	denoisedSE	non-chimeric	ASV Length Filter	ASV Remain
HV1	89,126	87,662	70,456	79.05%	69,967	67,004	66,917	75.08%
HV2	65,461	63,838	46,449	70.96%	44,575	44,160	44,160	67.46%
HV3	51,331	50,451	41,555	80.95%	41,388	41,388	40,475	78.85%
HV4	95,068	93,286	77,010	81.01%	76,620	75,820	75,322	79.23%
HV5	95,265	93,334	76,844	80.66%	76,528	75,066	74,895	78.62%
HV6	60,163	59,122	47,564	79.06%	45,656	45,102	45,102	74.97%
BR1	107,265	105,616	85,995	80.17%	85,830	85,830	85,782	79.97%
BR2	101,052	99,439	81,362	80.51%	81,083	81,083	81,061	80.22%
BR3	109,667	107,996	88,156	80.39%	87,451	83,664	83,563	76.20%
BR4	54,326	53,573	43,892	80.79%	43,767	43,574	43,436	79.95%
BR5	84,298	83,113	68,610	81.39%	68,301	66,518	66,408	78.78%
BR6	102,283	100,629	82,871	81.02%	82,541	82,360	82,154	80.32%
BR7	91,626	90,071	74,371	81.17%	74,104	74,104	74,072	80.84%
BR8	106,073	104,367	86,001	81.08%	85,678	85,678	85,590	80.69%
BR9	89,277	87,783	72,249	80.93%	71,773	70,781	70,703	79.20%
BR10	30,367	29,888	24,281	79.96%	24,156	24,156	23,906	78.72%
BR11	105,635	103,897	85,585	81.02%	85,211	84,265	84,265	79.77%
BR12	84,603	82,979	66,745	78.89%	66,135	66,135	66,093	78.12%
BR13	106,983	105,033	85,693	80.10%	85,067	85,067	84,548	79.03%
BR14	78,143	76,657	62,966	80.58%	62,666	62,403	62,369	79.81%
BR15	90,651	89,197	73,158	80.70%	72,389	68,462	68,138	75.17%
BR16	115,903	113,405	93,855	80.98%	93,465	93,465	93,277	80.48%
BR17	98,972	96,865	79,628	80.46%	79,324	79,324	79,310	80.13%
BR18	104,551	102,582	84,254	80.59%	83,833	83,833	83,785	80.14%
HD1	42,601	41,995	34,149	80.16%	33,808	33,808	33,500	78.64%
HD2	98,123	96,611	79,275	80.79%	78,552	76,912	76,906	78.38%
HD3	80,892	79,318	65,010	80.37%	64,556	63,525	63,234	78.17%
HD4	126,330	123,643	93,467	73.99%	90,259	89,888	89,885	71.15%
HD5	11,696	11,450	9,046	77.34%	8,636	8,411	8,310	71.05%
CV1	123,432	121,048	99,456	80.58%	99,150	96,916	96,877	78.49%
CV2	89,283	87,410	67,795	75.93%	65,980	65,192	63,970	71.65%

Table S3. Raw data statistics of shotgun metagenome sequencing, related to STAR Methods.

Sample ID	Total bases (bp)	Total reads	GC (%)	AT (%)	Q20 (%)	Q30 (%)
BR1	12,105,363,772	80,167,972	40.7	59.3	98.8	95.3
BR2	12,892,789,814	85,382,714	41.3	58.7	98.8	95.3
BR17	12,515,838,246	82,886,346	41.4	58.6	98.8	95.2
BR18	12,735,903,230	84,343,730	41.1	58.9	98.8	95.3
HV4	12,467,001,826	82,562,926	40.9	59.1	98.8	95.3

Table S4. QC statistics of shotgun metagenome sequencing, related to STAR Methods.

Sample	Raw reads	Trimmed reads	Host removal reads	QC Passed reads (%)
BR1	40,083,986	38,266,908	3,968	0.01%
BR2	42,691,357	40,759,432	4,016	0.01%
BR17	41,443,173	39,606,351	2,106	0.01%
BR18	42,171,865	40,186,337	4,037	0.01%
HV4	41,281,463	39,362,964	899	0.00%
